# Case Report: Genetic Analysis of PEG-Asparaginase Induced Severe Hypertriglyceridemia in an Adult With Acute Lymphoblastic Leukaemia

**DOI:** 10.3389/fgene.2022.832890

**Published:** 2022-02-14

**Authors:** Arcangelo Iannuzzi, Mario Annunziata, Giuliana Fortunato, Carola Giacobbe, Daniela Palma, Alessandro Bresciani, Emilio Aliberti, Gabriella Iannuzzo

**Affiliations:** ^1^ Department of Medicine and Medical Specialties, A. Cardarelli Hospital, Naples, Italy; ^2^ Division of Hematology, A. Cardarelli Hospital, Naples, Italy; ^3^ Department of Biochemistry and Medical Biotechnology, University of Naples “Federico II”, Naples, Italy; ^4^ Division of Gastroenterology, North Tees University Hospital, Stockton-on Tees, United Kingdom; ^5^ Department of Clinical Medicine and Surgery, Federico II University, Naples, Italy

**Keywords:** PEG-asparaginase, acute lymphoblastic leukaemia, hypertriglyceridemia, APOC3, genetic testing

## Abstract

PEG-Asparaginase (also known as Pegaspargase), along with glucocorticoids (predominantly prednisolone or dexamethasone) and other chemotherapeutic agents (such as cyclophosphamide, idarubicin, vincristine, cytarabine, methotrexate and 6-mercaptopurine) is the current standard treatment for acute lymphoblastic leukaemia in both children and adults. High doses of PEG-asparaginase are associated with side effects such as hepatotoxicity, pancreatitis, venous thrombosis, hypersensitivity reactions against the drug and severe hypertriglyceridemia. We report a case of a 28-year-old male who was normolipidemic at baseline and developed severe hypertriglyceridemia (triglycerides of 1793 mg/dl) following treatment with PEG-asparaginase for acute lymphoblastic leukaemia. Thorough genetic analysis was conducted to assess whether genetic variants could suggest a predisposition to this drug-induced metabolic condition. This genetic analysis showed the presence of a rare heterozygous missense variant c.11G > A-p.(Arg4Gln) in the APOC3 gene, classified as a variant of uncertain significance, as well as its association with four common single nucleotide polymorphisms (SNPs; c.*40C > G in APOC3 and c.*158T > C; c.162-43G > A; c.-3A > G in APOA5) related to increased plasma triglyceride levels. To our knowledge this is the first case that a rare genetic variant associated to SNPs has been related to the onset of severe drug-induced hypertriglyceridemia.

## Introduction

L-asparaginase has been used for over 40 years for the treatment of Acute Lymphoblastic Leukaemia (ALL), especially in paediatric patients, where the incidence of this disease is highest. It has been demonstrated that using L-asparaginase in adult patients improves prognosis and, when used at higher doses, its therapeutic effect is the same as in paediatric patients (Quist-Paulsen et al., 2020). It is worth remembering that L-asparaginase treatment can cause several side effects, which may require a suspension of treatment. Among the most frequently observed side effects are hepatotoxicity, pancreatitis, venous thrombosis (sometimes complicated by pulmonary emboli), and severe hypertriglyceridemia (Hoogerbrugge et al., 1997; Grace et al., 2011; Truelove et al., 2013; Oparaji et al., 2017; Finch et al., 2020a; Burke et al., 2020; Underwood et al., 2020; Chen et al., 2021; Kumar et al., 2021). Moreover, asparaginase therapy can cause hypersensitivity reactions and development of asparaginase-antibodies that limit the action of the drug and increase the risk of leukemia relapse. Clinical pharmacogenetics studies suggest that the family of nuclear factor of activated T-cells transcription factors could play a role in increasing the risk of hypersensitivity reactions due to L-asparaginase; genetic inhibition of these factors protects against asparaginase hypersensitivity, at least in mice (Woo et al., 1998; Fernandez et al., 2015; Rathod et al., 2019a; Rathod et al., 2019b; Rathod et al., 2020). There are three formulations of asparaginase: L-asparaginase, which is endogenously produced by *Escherichia coli*, Pegylated asparaginase (PEG-ASP) which is a pegylated form of L-asparaginase and results in a prolonged half-life and decreased immunogenicity and *Erwinia* asparaginase, which is derived from the bacterium *Erwinia* chrysanthemi, and is immunologically different from the *Escherichia coli* derived asparaginase forms and which could be used in patients allergic to other formulations. At present the pegylated long-acting form of L-asparaginase (PEG-ASP) is by far the most used therapeutic option in both adult and paediatric ALL patients due to a more prolonged effect, a reduced incidence of silent antibody and more rapid clearance of lymphoblasts than native asparaginase (Avramis et al., 2002). The aim of this work is to describe a clinical case of severe hypertriglyceridemia arising following treatment with PEG-asparaginase in a patient with ALL, and to verify whether or not a genetic profile leading to the onset of this serious complication can be identified.

### Case Description and Diagnostic Assessment

**FIGURE 1:**
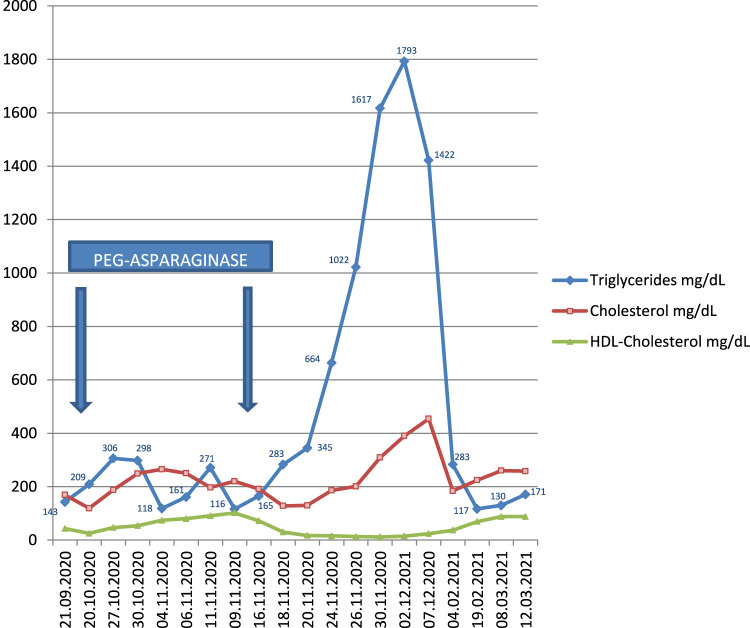
Timeline graphic showing Triglycerides, Total Cholesterol and HDL-Cholesterol concentration during therapy for acute lymphoblastic leukemia in a 28 years man following Ginema LAL 1913 protocol (first 4 cycles). [Numbers in blue represent concentrations of triglycerides at different interval times].

A 28-year-old male, with Ph negative ALL underwent chemotherapy according to the GIMEMA LAL 1913 protocol (approved by the Cardarelli Hospital Etichal Committee) and achieved complete remission with minimal residual disease (MRD), while both immunophenotyping by flow cytometry and molecular testing yielded a negative result. This protocol involves the administration of PEG-ASP during induction/consolidation (cycles 1, 2, 5, and 6), in addition to other agents, including prednisone, dexamethasone and other chemotherapeutic agents (such as cyclophosphamide, idarubicin, vincristine, cytarabine, methotrexate and 6-mercaptopurine). On admission to hospital (31 August 2020) the blood pressure was 150/90 mmHg; heart rate 124 beats per minute and the cardiologist prescribed bisoprolol fumarate. On physical examination mildly enlarged lymph nodes were palpable in latero-cervical chains and in the left axilla. Palpation of the abdomen revealed mild hepatomegaly and splenomegaly. His family history was noteworthy for breast cancer in his mother. Laboratory Data: Glucose was 93 mg/dl; triglycerides 116 mg/dl; cholesterol 220 mg/dl; HDL-cholesterol 102 mg/dl; amylase 38 IU/L; lipase 26 IU/L; lactate dehydrogenase 1169 IU/L; alanine transaminase 164 IU/L; aspartate transaminase 71 IU/L; gamma-glutamyl transpeptidase 339 IU/L; C-Reactive Protein 57 mg/L; Hematocrit 40%; Hemoglobin 14.2 g/dl; Platelets 101.000/mm^3^; White-cell count 33.380/mm^3^ (Differential count: Neutrophils 13.7%; Lymphocytes 75.6%; Monocytes 7.5%; Eosinophils 0.6%; Basophils 2.6%). A CT-study showed bilateral cervical and left axillary lymphadenopathy (max 1 cm), mild hepatomegaly and splenomegaly. Following the second round of treatment with PEG-ASP he was found to have: glucose 83 mg/dl; **triglycerides 1793 mg/dl**; cholesterol 390 mg/dl; HDL-cholesterol 15 mg/dl; amylase 32 IU/L; lipase 23 IU/L; lactate dehydrogenase 270 IU/L; alanine transaminase 44 IU/L; aspartate transaminase 20 IU/L; gamma-glutamyl transpeptidase 126 IU/L. There were no signs, symptoms, or investigations suggestive of acute pancreatitis. PEG-ASP was no longer administered to the patient. The nutritionist prescribed a hypocaloric, higher-protein, lower carbohydrate diet, and the internist prescribed omega-3 fatty acids and a statin. At follow-up his lipid profile was normal. Figure 1 shows concentrations of triglycerides, total cholesterol, HDL-cholesterol at different interval times during chemotherapy.

In May 2021 the patient had a relapse of ALL and was treated for refractory acute lymphoblastic leukemia with high dose cytosine arabinoside and mitoxantrone (HAM).

In August 2021: the patient moved to another city in North Italy and was lost to follow-up.

### Genetic Screening

Genomic DNA was extracted from peripheral blood and genetic screening was performed using a custom panel of 16 genes, associated to severe hypertriglyceridemia: APOA1, APOA4, APOA5, APOC1, APOC2, APOC3, APOC4, APOE, ANGPTL3, LPL, GPIHBP1, LMF1, GPD1, CREB3L3, GCKR, LRP1. For each gene the coding regions, 25 bp in each of the intronic boundaries, the 5′UTR and the 3′UTR regions, were included. Genomic libraries were obtained using the SureSelect Target Enrichment protocol (Agilent Technologies) and the high throughput sequencing was performed using Illumina technology (PE 2 × 150 bp). Data analysis was carried out using Alissa Interpret Rev.5.2.6 software (Agilent Technologies). Variants were firstly filtered on their minor allele frequency (MAF), and pathogenicity of rare prioritized variants (with a MAF <1%) was assessed according to the American College of Medical Genetics and Genomics (ACMG) guidelines (Richards et al., 2015). All the prioritized variants were also validated by Sanger sequencing. The Multiplex Ligation-dependent Probe Amplification was also performed to verify the presence of rearrangements such as deletions and/or duplications in the coding region of the lipoprotein-lipase (LPL) gene (Giacobbe et al., 2020). Genetic analysis in this patient showed no evidence of variants causing severe familial hypertriglyceridemia. No common LPL gene variants (Asp9Asn, Asn291Ser, Trp86Arg, Gly188Glu, Pro207Leu, Asp250Asn) associated with the increase of plasma triglyceride levels were highlighted (Gehrisch, 1999). However, a rare missense variant, c.11G > A–p.(Arg4Gln), in the *APOC3* gene and four single nucleotide polymorphisms (SNPs) associated with increased plasma triglyceride levels at heterozygous state, were found. The APOC3 rare variant was classified as of uncertain clinical significance based on the ACMG guidelines. Genetic profile of patient is shown in Table 1.

**TABLE 1:** Variants identified in this patient.

Nucleotide substitution	Amino acid substitution	dbSNP^1^ code	MAF^2^ (%)	Genotype^3^	HGMD database^4^	Phenotype^5^	Variant classification^6^	PMID^7^
*APOC3* (NM_000040.3)								
c.11G > A	p.(Arg4Gln)	rs779597455	0.002	Hz	—	—	Uncertain significance	—
c.*40C > G	—	rs5128	13	Hz	CR041556	Increased plasma triglyceride levels	Benign	25928461
*APOA5* (NM_052968.5)								
c.*158T > C	—	rs2266788	6.3	Hz	CR014773	Increased plasma triglyceride levels	Benign	11588264
								24387992
c.162-43G > A	—	rs2072560	5.8	Hz	CS014761	Increased plasma triglyceride levels	Benign	11588264
c.-3A > G	—	rs651821	10	Hz	CR080753	Increased plasma triglyceride levels	Benign	28008009
								24387992

1 www.ncbi.nlm.nih.gov

2 MAF: minor allele frequency; gnomad.broadinstitute.org.

3 Hz: Heterozygous.

4 Human Gene Mutation Database (HGMD^®^ Professional 2021.3).

5 Clinical phenotype associated with the SNP.

6 American College of Medical Genetics and Genomics (ACMG; Richards et al. Genet Med 2015).

7 PMID: PubMed ID references.

## Discussion

Glucocorticoids and PEG-ASP are fundamental treatments for ALL in both adults and children. However, these therapies, especially when combined, can result in side effects which include alteration of the lipid profile, particularly severe hypertriglyceridemia. The mechanism by which these drugs lead to the onset of hypertriglyceridemia has not yet been elucidated. Glucocorticoids increase the synthesis of triglycerides and mobilise fatty acids, while activating lipoprotein lipase (LPL), an enzyme responsible for the hydrolysis of triglycerides (Peckett et al., 2011). PEG-ASP, on the other hand, seems to inhibit LPL activity (Hoogerbrugge et al., 1997). Therefore, when glucocorticoids are given simultaneously with PEG-ASP there is an increase in triglyceride synthesis without an increase in hydrolysis. In children the frequency of hypertriglyceridemia following treatment with corticosteroids and PEG-ASP for ALL is between 4 and 19%, depending on the protocol followed (Steinherz, 1994; Parsons et al., 1997; Cohen et al., 2010; Bhojwani et al., 2014). In young adults, as in our case, the frequency of severe hypertriglyceridemia is around 11% (Stock et al., 2019). Among the disorders of lipid metabolism, the genetic spectrum for familial hypercholesterolemia and its associated clinical implications is well established (Romano et al., 2010; Di Taranto et al., 2021). The relationship between severe hypertriglyceridemia and its clinical implications is less evident, whereas there is a well documented relationship with acute pancreatitis (Scherer et al., 2014). In patients with ALL the association between severe hypertriglyceridemia and pancreatitis is rarely seen (Tong et al., 2014). Severe hypertriglyceridemia, in the form of Familial Chylomicronaemia Syndrome (FCS), is a rare autosomal recessive disease caused by pathogenic variants especially in the LPL gene but also in APOC2, APOA5, LMF1 and GPIHBP1. Recently, it has been hypothesised that severe hypertriglyceridemia could be more frequently caused by the coexistence of rare variants in genes recognized in FCS, as well as by the presence of common or rare variants currently not recognized in the metabolism of triglycerides. This condition has been named multifactorial chylomicronaemia syndrome (MCS) (Dron and Hegele, 2020; Paquette et al., 2021). MCS represents a predisposing condition which could expose the patients to a 4 times greater risk of developing severe hypertriglyceridemia (Dron et al., 2019). Patients with the same heterozygous changes in the same genes who have normal or only slightly elevated triglyceride values, were also described, suggesting that these variants are only partially penetrating. Most variants interfering with triglyceride metabolism and causing hypertriglyceridemia are loss of function variants (LOF), and mainly found in the LPL, APOC2, APOA5, LMF1 and GPIHBP1 genes. Recently a gain of function (GOF) variant, in the APOC3 gene has been described in patients with severe hypertrigliceridemia (Sundaram et al., 2017). This APOC3 gene encodes a protein component of VLDL and chylomicrons and can inhibit lipoprotein lipase enzyme activity. Elevated plasma levels of APOC3 are also considered as a major risk factor for hypertriglyceridemia (Witztum et al., 2019). In light of the above the question we asked ourselves was: why, with equal doses of PEG-ASP, severe hypertriglyceridemia only occurs in a limited number of cases, even if these patients have a normal lipid profile at baseline? We can hypothesize that there are genetic variants which affect the onset of severe hypertriglyceridemia after certain treatments, for example with PEG-ASP. It is possible that non-genetic factors, such as the use of some drugs, could result in the development of severe hypertriglyceridemia in an individual who is predisposed to hypertriglyceridemia due to being a carrier of a gene with an increased associated risk. In a previous paper, Finch et al. found no significant genetic predisposition to hypertriglyceridemia in patients with ALL treated with PEG-ASP (Finch et al., 2020b). In our clinical case we identified a heterozygous variant, associated with triglyceride metabolism and potentially implicated in the origin of severe hypertriglyceridemia induced by PEG-ASP. To the best of our knowledge, this is the first case in which a potentially triglyceride-raising genetic variant has been detected in a patient with normal lipids at baseline and subsequent severe hypertriglyceridemia and hypercholesterolaemia following treatment with PEG-ASP. To date the APOC3 variant c.11G > A–p.(Arg4Gln) found in our patient and classified as of uncertain clinical significance according to ACMG guidelines, has never been described as associated to severe hypertriglyceridemia and if its pathogenicity resulted as GOF, the patient would be considered as carrier of a rare pathogenic variant.

Polymorphisms in the *APOC3* and *APOA5* genes, from the APOA1/APOC3/APOA4/APOA5 gene cluster on chromosome 11q23, have been associated with interindividual variation in plasma triglycerides. However, the degree to which polymorphisms in the *APOC3* and *APOA5* genes can be independently associated with triglyceride levels remains to be determined (Hallman et al., 2006). The close genomic locations of these two genes as well as their functional similarity have hindered efforts to define whether each gene independently influences human triglyceride concentrations. Several studies reported the existence of a common haplotype in the APOAV region influencing plasma triglyceride levels in some race-specific populations (Lai et al., 2003; Lai et al., 2004; Klos et al., 2005). Three APOA5 SNPs identified in our patient (rs651821, rs2072560 and rs2266788) were described by Dror et al. as associated with another SNP (rs662799) in significant linkage disequilibrium as well as with a 27–38% increase in triglyceride concentration in three ethnic groups (Ken-Dror et al., 2010). A large meta-analysis highlighted the association of the APOC3 rs5128 polymorphism with highly increased triglyceride plasma levels (Song et al., 2015).

Furthermore, Olivier et al. demonstrated that the APOA5 locus is separated from the other apolipoprotein genes by a region of increased recombination, thereby supporting the idea that APOA5 represents an independent risk gene affecting plasma triglyceride concentrations in humans (Olivier et al., 2004). The presence of the *APOC3* rare variant c.11G > A–p.(Arg4Gln) together with the common SNPs associated with high levels of triglycerides [c.*40C > G in *APOC3* (NM_000040.3) and c.*158T > C; c.162-43G > A; c.-3A > G in *APOA5* (NM_052968.5)] could lead to classify our patient as suffering from MCS.

This latter condition could be considered as a predisposing factor of developing a hypertriglyceridemic phenotype after PEG-ASP treatment. Of course, a single case cannot demonstrate with any certainty whether there is a genetic cause at the root of severe hypertriglyceridemia induced by drugs. However, this could encourage further research into genes less frequently associated with the phenotypic manifestation of severe hypertriglyceridemia that could play a significant role as co-factors responsible for some forms of hypertriglyceridemia.

Strengths and limitations: The principal strength of this paper is the original finding (to our knowledge for the first time in the literature) of a potentially triglyceride-raising genetic variant implicated in the origin of severe hypertriglyceridemia induced by PEG-ASP in a patient with normal lipids at baseline. Moreover, this study highlights that the use of a custom capture-based target enrichment NGs panel -containing 16 genes to date known as being associated with Familial Chylomicronemia Syndrome and severe hypertriglyceridemia-allowed us to find a clear genetic pattern characterized by a rare variant in the apo-C3 gene together with four common SNPs that could be related to the onset of severe drug-induced Hypertriglyceridemia. A limitation of the present study is that no mechanistic study supports our findings and we can conclude that genetic variants could be considered as a predisposing factor of developing a hypertriglyceridemia and may not be solely responsible for severe hypertriglyceridemia after treatment with PEG-ASP.

## Data Availability

The datasets for this article are not publicly available due to concerns regarding participant/patient anonymity. Requests to access the datasets should be directed to the corresponding author.
